# Strawberry fruit skins are far more permeable to osmotic water uptake than to transpirational water loss

**DOI:** 10.1371/journal.pone.0251351

**Published:** 2021-05-13

**Authors:** Grecia Hurtado, Eckhard Grimm, Martin Brüggenwirth, Moritz Knoche

**Affiliations:** Institute for Horticultural Production Systems, Leibniz-Universität Hannover, Hannover, Germany; Universidade do Minho, PORTUGAL

## Abstract

Water movements through the fruit skin play critical roles in many disorders of strawberry (*Fragaria × ananassa* Duch.) such as water soaking, cracking and shriveling. The objective was to identify the mechanisms of fruit water loss (dry skin, transpiration) and water uptake (wet skin, osmosis). Fruits were held above dried silica gel or incubated in deionized water. Water movements were quantified gravimetrically. Transpiration and osmotic uptake increased linearly with time. Abrading the thin cuticle (0.62 g m^-2^) increased rates of transpiration 2.6–fold, the rates of osmotic uptake 7.9-fold. The osmotic potential of the expressed juice was nearly the same for green and for white fruit but decreased in red fruit stages. Fruit turgor was low throughout development, except for green fruit. There was no relationship between the rates of water movement and fruit osmotic potential. The skin permeance for transpiration and for osmotic uptake were both high (relative to other fruit species) but were two orders of magnitude greater for osmotic uptake than for transpiration. Incubating fruit in isotonic solutions of osmolytes of different sizes resulted in increases in fruit mass that depended on the osmolyte. The rate of osmotic uptake decreased asymptotically as molecular size of the osmolyte increased. When transpiration and osmotic uptake experiments were conducted sequentially on the same fruit, the rates of transpiration were higher for fruit previously incubated in water. Fluorescence microscopy revealed considerable microcracking in a fruit previously incubated in water. Our findings indicate that the high permeance for osmotic uptake is accounted for by an extremely thin cuticle and by viscous water flow through microcracks and along polar pathways.

## Introduction

Strawberry is a highly perishable commodity. The quality of strawberries at retail is often compromised by pre- and postharvest factors. Preharvest factors include the exposure of fruit to rain in the course of growth and development. Classical disorders related to rain exposure are fruit cracking and water soaking [1]. Both disorders are often followed by fruit rot, such as grey mold. During the harvesting and subsequent postharvest storage, transport and handling, fruit water loss due to transpiration is critical. Ultimately it results in shrivel and in compromised appearance. In addition, the mass loss that occurs along the marketing chain requires fruit containers to be ‘over-packed’ so as to ensure a pre-specified weight for the consumer. Compromised fruit quality and overpacking of fruit containers both cause significant financial loss.

Water movement through the fruit surface is likely to be a critical factor in all the above disorders. Although the mechanistic bases of strawberry fruit cracking and water soaking have not been investigated in great detail, research on similar disorders in other fruitcrop species indicates the involvement of water uptake directly through the fruit surface and, possibly also of water uptake through the fruit vascular system [2]. Such research includes the rain cracking of soft, fleshy fruits such as apples (*Malus × domestica* Borkh.) [2,3], blueberries (*Vaccinium corymbosum* L.) [4], plums (*Prunus domestica* L.) [5], tomatoes (*Solanum lycopersicum* L.) [6], citrus lemon (*Citrus* ×*limon* (L.) Burm. f.) [7], grape berries (*Vitis vinifera* L.) [8], sweet cherries (P*runus avium* L.) [9], jostaberry (*Ribes nidigrolaria* B.), gooseberry (*Ribes uva-crispa* L.), and black currant (*Ribes nigrum* L.) [10]. For strawberry fruit, water loss through the fruit skin is likely to be a critical factor in shriveling and compromised appearance. For sweet cherry, shrivel-type phenomena such as ‘orange peel’ disorder are caused by skin dehydration, with the latter exacerbated if fruit are allowed to transpire excessively [11–13].

The above arguments indicate that water loss through the strawberry fruit surface by transpiration (in dry air) and water uptake by osmosis (when the fruit surface is wetted) are likely to be the major determinants in the shriveling, cracking and water soaking of strawberries. Little is known about water movement through the strawberry fruit skin. It is worth noting that a strawberry ‘fruit’ has an unusual morphology. It is primarily comprised of receptacle tissue—a strawberry is a pseudocarp or false fruit, not a true fruit comprised of mostly pericarp tissue. The actual fruits of a strawberry are the tiny achenes embedded in the surface of an expanded receptacle. The strawberry is also unusual in that it develops over quite a short period of time, suggesting that the fruit skin is subjected to particularly high rates of strain. A better understanding of these underlying factors should be helpful in developing improved strategies for strawberry breeding, cultivation and handling so as to reduce or eliminate fruit quality impairments.

The objective of this study is to identify the mechanism(s) of water movement in transpiration (loss in dry air) and osmosis (uptake when wetted) through the surface of detached strawberry fruit.

## Materials and methods

### Plant material

Strawberry fruit were harvested from commercial plantings at Gleidingen, Bad Nenndorf, research plots of the Horticultural Research Center in Cologne-Auweiler and from the green house and growth chamber facility at the Campus Herrenhausen of Leibniz University, Hannover, Germany. Temperature and relative humidity (RH) of the growth chamber were set at 20/16°C and 60/80% RH during a 16 h day/night photoperiod.

Unless specified otherwise, fruit were harvested randomly and at commercial ripeness (>80% of the fruit surface red). Fruit of the same size, shape and color and free of visible defects were selected. Fruit was processed fresh on the day of sampling or held at 2°C and 80% RH for no longer than 2 d. Previous studies showed that holding fruit for up to 2 d under these conditions had no effect on rates of water uptake or transpiration. Unless otherwise specified, the calyx was removed from the fruit by carefully pulling and the resulting hole sealed using a fast-curing silicone rubber (Silicone rubber, SE 9186 Clear; Dow Corning Corp., Midland, USA). The number of individual fruit replicates was 15 unless otherwise specified.

### General procedure

For transpiration experiments, fruits were incubated for 1.5 h in a polyethylene (PE) box, usually above dry silica gel (RH~0%; [14]) and weighed individually at 30-min intervals. The rate of transpiration (F_t_; mg h^-1^) was calculated on an individual fruit basis as the slope of a linear regression line fitted through a plot of fruit mass versus time.

For osmotic uptake experiments, fruits were incubated individually in deionized water for 1.5 h. Osmotic uptake was determined gravimetrically. Fruits were carefully blotted using soft tissue paper and then weighed at 30-min intervals. The rate of osmotic uptake was calculated (F_f_; mg h^−1^) on an individual fruit basis from the slope of a linear regression line fitted through a plot of fruit mass versus time.

All experiments were carried out in a temperature controlled laboratory at 22°C.

### Experiments

The time courses of osmotic uptake and transpiration were established in ‘Clery’ fruits. To ensure the repeated handling and blotting of fruit in the osmotic uptake experiment did not damage the fruit surface, rates of osmotic uptake of fruit blotted and weighed multiple times (at 0, 0.5, 1, 1.5 and 3 h) and of fruit blotted and weighed just once (at 3 h) were compared.

The effect of the cuticle on water movement was studied by abrading the cuticle from the fruit surface of ‘Florentina’ using sand paper (grain 400). Non-treated fruit served as control. Weighing intervals were modified in water uptake assays to avoid bursting of cells and leakage of osmolytes (osmotic water uptake) and excessive dessication (transpiration). Osmotic uptake was determined at 1 min intervals for up to 3 min for fruit with an abraded cuticle. Control fruit was measured at intervals of 5 min for up to 15 min.

The effects of fruit size were investigated by selecting ‘Clery’ fruit of differing mass. Fruit surface area was calculated from fruit dimensions determined from calibrated photographs (Lumix DMC-G80; Panasonic Corporation, Osaka, Japan) by image analysis (cellSens Dimension 1.7.1; Olympus Soft Imaging Solutions, Münster, Germany). Briefly, the fruit was assumed to represent a truncated cone capped by two halves of rotational prolate ellipsoids. Using this approximation fruit surface area (A) was calculated from the upper and lower diameters of the cone, cone height and the heights of the two rotational prolate ellipsoids, one on either end. The flow rates of transpiration (F_t_) and osmotic uptake (F_f_) were quantified as described above. The flux densities (kg m^-2^ s^-1^) of water in transpiration (J_t_) and in osmotic uptake (J_f_) were calculated by dividing the transpiration flux and osmotic uptake flux by the corresponding fruit surface area.

The effects of juice osmotic potential on osmotic water uptake and transpiration were studied using ‘Florentina’ fruit of similar mass (mean 11.7 ± 0.1 g, range: 10.3–13.5 g) harvested at commercial ripeness, as indexed by color. Rates of osmotic uptake and transpiration were measured as described above. The osmotic potential of the expressed juice was determined by water vapor pressure osmometry (VAPRO 5600; Wescor, Utah, USA) on an individual fruit basis. The flow per unit osmotic potential was calculated by dividing F_t_ and F_f_ by the osmotic potential. This procedure normalizes for differences in driving force.

The effect of fruit development stage on transpiration and on osmotic uptake was studied in ‘Clery’ strawberry. Fruit growth was followed gravimetrically. Digital photographs were taken and the fruit surface area quantified from fruit dimensions using the model described above. The changes in color (CM-2600 d, orifice 3 mm diameter; Konica Minolta, Tokyo, Japan) and in osmotic potential of the expressed juice (VAPRO 5600; Wescor, Utah, USA) were monitored at 5-d intervals until fully ripe, beginning at 8 d after full bloom (DAFB). Rates of transpiration and osmotic uptake were determined. The skin permeances in transpiration and in osmotic uptake were calculated as described below.

The fruit water potential (Ψ_fruit_) was also determined using water-uptake experiments. ‘Florentina’ fruit were incubated in mixed solutions of increasing concentrations of fructose and glucose in equal molar ratios. These two monosaccharides represent the most abundant osmolytes in strawberry juice and together account for 63.5% of the osmolytes present [15]. Osmolarities of the incubation solutions were 0, 250, 500, 750 and 1000 mmol kg^-1^. The time courses for the osmotic uptake experiment were established at 0, 0.5, 1, 1.5 and 30 h for all hypertonic solutions (≥500 mmol kg^-1^). For the hypotonic solutions, i.e. the water control and the solution of 250 mmol kg^-1^, incubation was terminated after 1.5 h–longer incubations led to extensive fruit cracking. Rates of osmotic uptake were calculated for each incubation interval (0 to 0.5 h, 0.5 to 1 h, 1 to 1.5 h and 1.5 h to 30 h). A linear regression was fitted through a plot of the rate of uptake during each interval versus the osmotic potential of the incubation solution. From the regression parameters obtained, the hypothetical osmotic potential of a solution that would result in zero change in fruit mass was calculated. At this null point the Ψ_Π_ of the incubation solution would exactly equal the fruit water potential (Ψ_fruit_) and a driving force for net water uptake is absent [16].

The effect of the molecular size of the osmolytes on net osmotic uptake into ‘Florentina’ strawberry was established by preparing incubation solutions at osmolarities that were isotonic to the Ψ_Π_ of juice expressed from the same batch of fruit. The osmolytes and their molecular masses were glycerol (92 g mol^-1^), glucose (198.2 g mol^-1^), sucrose (342.3 g mol^-1^), polyethylene glycol (PEG) 1500 (1500 g mol^-1^) and PEG 6000 (6000 g mol^-1^).

The relationship between osmotic uptake and transpiration was studied by sequentially incubating ‘Laetitia’ fruit, first in water and then above silica gel and vice-versa. Thereafter, fruit were incubated in acridine orange (0.1%) (Carl Roth, Karlsruhe, Germany) for 5 min, then rinsed with deionized water and carefully blotted. The fruit surface was then inspected under incident white light and incident fluorescent light using a binocular microscope (Leica MZ10F with filter GFP plus 480–440 nm excitation, ≥510 nm emission; Leica Microsystems GmbH, Wetzlar, Germany).

### Water potential, osmotic potential and turgor

To quantify fruit water potential (Ψ), the osmotic potential (Ψ_Π_) and turgor (Ψ_p_) were established in developing ‘Clery’ fruit grown in a growth chamber. The developmental stage of a fruit was indexed by the change in fruit color (CM-2600 d; Konica Minolta, Tokyo, Japan). Fruit was held at 4°C for a maximum of 1 h before turgor measurement. The Ψ_Π_ was determined by water vapor pressure osmometry (VAPRO 5600; Wescor, Utah, USA) from the juice expressed using a garlic press. The Ψ_p_ of the cells of the outer flesh were determined using a cell pressure probe (CPP; [17,18]). The capillary was carefully inserted into the cells (< 0.5 mm below the fruit surface) under a horizontal microscope. Following volume correction, the peak pressure of the system was recorded. This pressure was taken as an estimate of Ψ_p_. For a detailed description of the protocol the reader is referred to [18]. The water potential of a cell was calculated as the algebraic sum of the osmotic potential (negative) and the turgor (positive).

### Calculating the permeances for osmotic water uptake and transpiration

The skin permeance for transpiration (P_t_; m s^-1^) was calculated as described earlier using flow rates determined in ‘Clery’, ‘Florentina’ and ‘Laetitia’ strawberry [19]. The P_t_ was calculated from the rate of transpiration (F_t_; kg s^-1^) divided by the product of the fruit surface area (A; m^2^), the density of water (ρ_w_; kg m^-3^) and the driving force for transpiration. In analogy to [20], the gradient in water activity (Δɑ_w_;dimensionless) across the fruit skin was used as the driving force (Eq 1). Because the humidity above dry silica is practically zero, Δɑ_w_ equals the water activity of the strawberry juice, which is approximately one.

$$P_{t}=\frac{F_{t}}{A_{fruit}∙\rho_{w}∙\Delta a_{w}}$$ (1)

The permeance for the opposite process of osmotic water uptake (P_f_, m s^-1^) was determined on the same cultivars using Eq 2. An alternate expression for the permeance in osmotic water uptake is the filtration permeability [21]. In Eq 2, F_f_ represents the rate of osmotic uptake, A the fruit surface area, and ΔΨ (MPa) the difference in water potential between the water potential of the fruit (Ψ_fruit_) and that of the incubation solution(Ψ_Π_). For fruit incubated in water (Ψ_Π_ = 0) the driving force for osmotic uptake is essentially equal to Ψ_fruit_. For mature fruit, Ψ_fruit_ was approximately equal to the osmotic potential of the expressed juice of the fruit (Ψ_Π_). The value of Ψ_Π_ was determined by water vapor pressure osmometry (VAPRO 5600; Wescor, Utah, USA) following expression of the juice using a garlic press. The parameters R, T, V_w_ and ρ_w_ are all constants where R (m^3^ MPa mol^-1^ K^-1^) represented the universal gas constant, T (K) the absolute temperature, V_w_ (m^3^ mol^-1^) the molar volume of water and ρ_w_ (kg m^-3^) the density of water. The permeance estimates P_t_ and P_f_ so obtained are directly comparable [19,22].

$$P_{f}=\frac{F_{f}}{A_{fruit}∙\Delta \Psi }∙\frac{RT}{\rho ∙\bar{V_{w}}}$$ (2)

### Mass of cuticle, cutin and wax

Mass of cuticle, cutin and wax was determined in ‘Florentina’ strawberries. Epidermal segments comprising cuticle, epidermis, and some adhering flesh were excised using a biopsy punch (6 mm diameter; Kai Europe, Solingen, Germany). The cuticular membrane (CM) was enzymatically insolated [23] by incubating in 50 mM citric acid buffer containing pectinase (90 ml l^-1^; Panzym Super E flüssig, Novozymes A/S, Krogshoejvej, Bagsvaerd, Denmark) and cellulase (5 ml l^-1^; Cellubrix L; Novozymes A/S) at room temperature. To prevent microbial growth, NaN_3_ was added at a final concentration of 30 mM. The isolated CMs were carefully cleaned from adhering cellular debris using a soft, camel-hair brush and desorbed in deionized water. Achenes were manually removed. Samples of CMs (n = 10) were dried above silica gel for 48 h and weighed. Subsequently, CMs were extracted by incubation in CHCl_3_/MeOH (1:1, v/v) for 24 h at room temperature. The dewaxed CMs (DCMs) were dried above silica gel for 48 h and their mass determined. CM mass per unit fruit surface area were calculated. The wax mass per unit area was calculated by subtracting the cutin mass per unit area from the cuticle mass per unit area. The experiment was carried out using 12 replicates.

### Data analyses

All experiments were conducted and analyzed using completely randomized designs. Data were analyzed by analysis of variance and linear regression. Means were compared using Tukey’s studentized range tests (p < 0.05) using R (version 3.5.1; R Foundation for Statistical Computing, Vienna, Austria). Unless individual observations are shown, i.e. Figs 2–4, 5C and 9A, data are presented as means ± standard errors. All data shown in figures and tables are available in the S1 Dataset.

## Results

**Fig 1:**
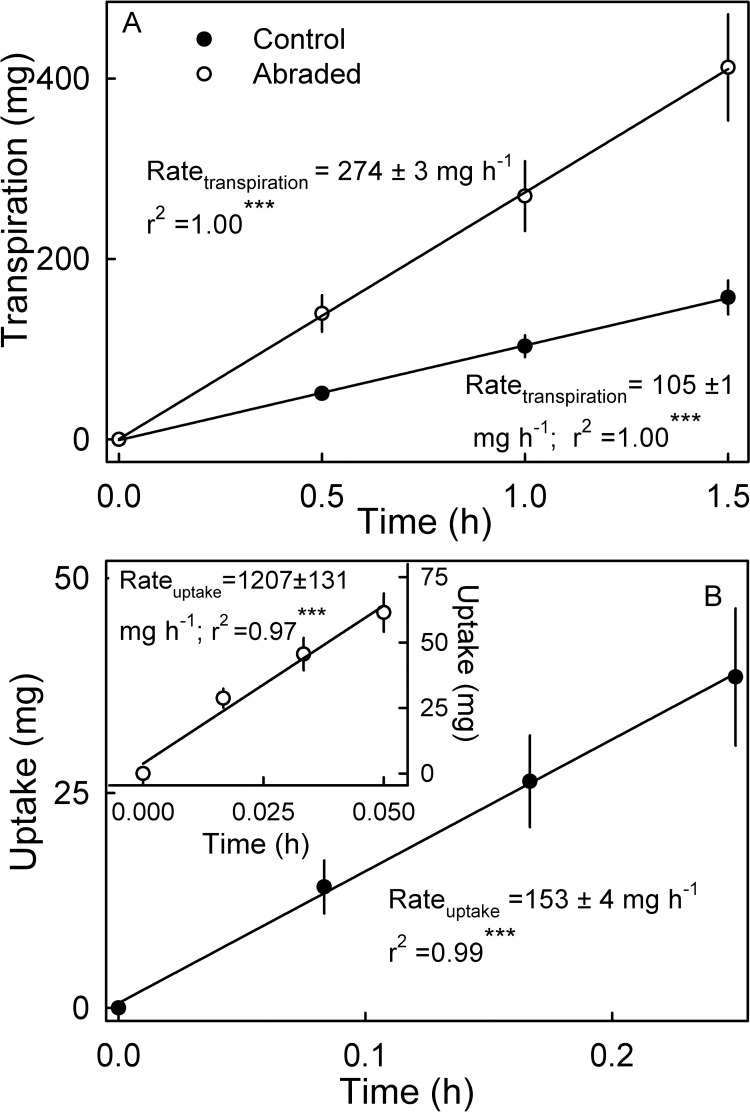
Effect of abrading the cuticle on water movement. (A) Transpiration and (B) osmotic water uptake. Significance of coefficients of determination (r^2^) at P < 0.001 indicated by ***.

Osmotic water uptake rate and transpiration rate through the surface of mature strawberries increased linearly with time (Fig 1). There were no significant differences between the rates of osmotic uptake of fruit that were repeatedly weighed and blotted (91.8 ± 13.6 mg h^-1^) and fruit that were weighed and blotted only once (124.8 ± 13.9 mg h^-1^; P < 0.115). Abrading the cuticle increased the amount of water transpired or taken up osmotically compared to the control (Fig 1). The strawberry cuticle was extremely thin as indicated by a low mass per unit area of cutin and wax (Table 1). The wax content averaged 19.2%.

**Table 1:** Mass of the cuticular membrane (CM), dewaxed CM (DCM) and permeances for transpiration (P_t_) and for osmotic uptake (P_f_) of a range of ripe fruitcrop species.

Species	Cultivar	Mass per unit area (g m^-2^)			P_t_ (x 10^−9^ m s^-1^)	P_f_ (x 10^−9^ m s^-1^)	Ratio P_f_/P_t_
		CM	DCM	Wax			
Strawberry	Clery	-	-	-	4.9±0.1	1108.0±86.1	226
	Florentina	0.62±0.02	0.50±0.01	0.12±0.01	4.4±0.2	863.4±37.7	196
	Laetitia	-	-	-	6.4±0.3	860.1±68.1	134
Grape [19,24]	Chardonnay	3.90±0.07	-	-	2.2±0.1	7.7±0.9	4
	Müller-Thurgau	3.30±0.03	-	-	2.5±0.1	8.9±1.5	4
	Riesling	4.56±0.04	3.21±0.09	1.35±0.08	1.6±0.1	4.1±1.2	3
Cherry [22,25]	Sam	1.31±0.04	0.94±0.04	0.37±0.01	1.8±0.2	44.5±19.1	25
	Hedelfinger	1.20±0.04	0.92±0.0	0.28±0.02	3.1±1.1	135.3±3.0	44
	Adriana	1.09±0.02	0.73±0.02	0.36±0.02	2.2±0.6	30.7±2.8	14
Tomato [22]	Sun Rise	8.08±0.30	-	-	1.0±0.2	15.2±3.2	15
Black currant [10]	Zema	5.04±0.04	4.09±0.03	0.95±0.01	-	77.0±4.0	-
Gooseberry [10]	Rote Triumph	5.62±0.08	5.03±0.08	0.59±0.00	-	52.0±1.0	-
Jostaberry [10]	Jostine	4.85±0.05	3.81±0.04	1.04±0.01	-	33.0±3.0	-

**Fig 2:**
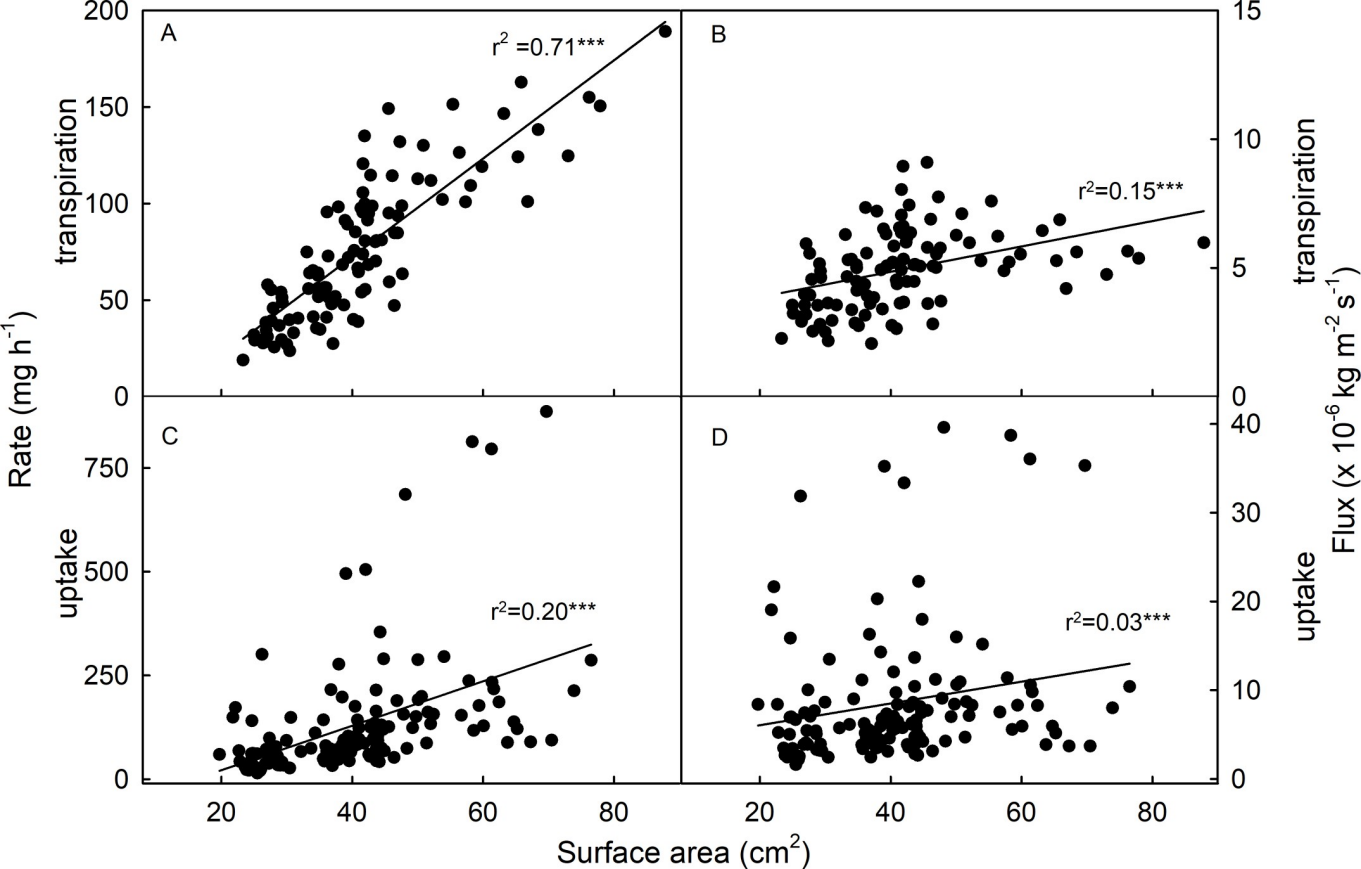
Relationship between fruit surface area and water movement. (A) Flow rates and (B) flux densities of transpiration from strawberry fruit. (C) Flow rates and (D) flux densities of osmotic uptake into strawberry fruit. Significance of coefficients of determination (r^2^) at P < 0.001 and indicated by ***.

Not surprisingly, the flow rates (mass of water, per hour, per fruit) for transpiration and for osmotic uptake were positively and significantly related to fruit surface area. But this surface-area relationship was closer for transpiration than for osmotic uptake, as indexed by a higher coefficient of determination for transpiration (Fig 2A and 2C). Also, not surprisingly, dividing these water flow rates by the corresponding fruit surface areas revealed that the water flux densities (mass of water, per hour, per unit area of fruit surface) for transpiration and for osmotic uptake were markedly less dependent on fruit surface area (Fig 2B and 2D). For a representative dataset, there was no significant correlation between fruit size and fruit osmotic potential (r = 0.01^ns^). This indicates the absence of a confounding interaction between size and osmotic potential for fruit of the same color maturity.

**Fig 3:**
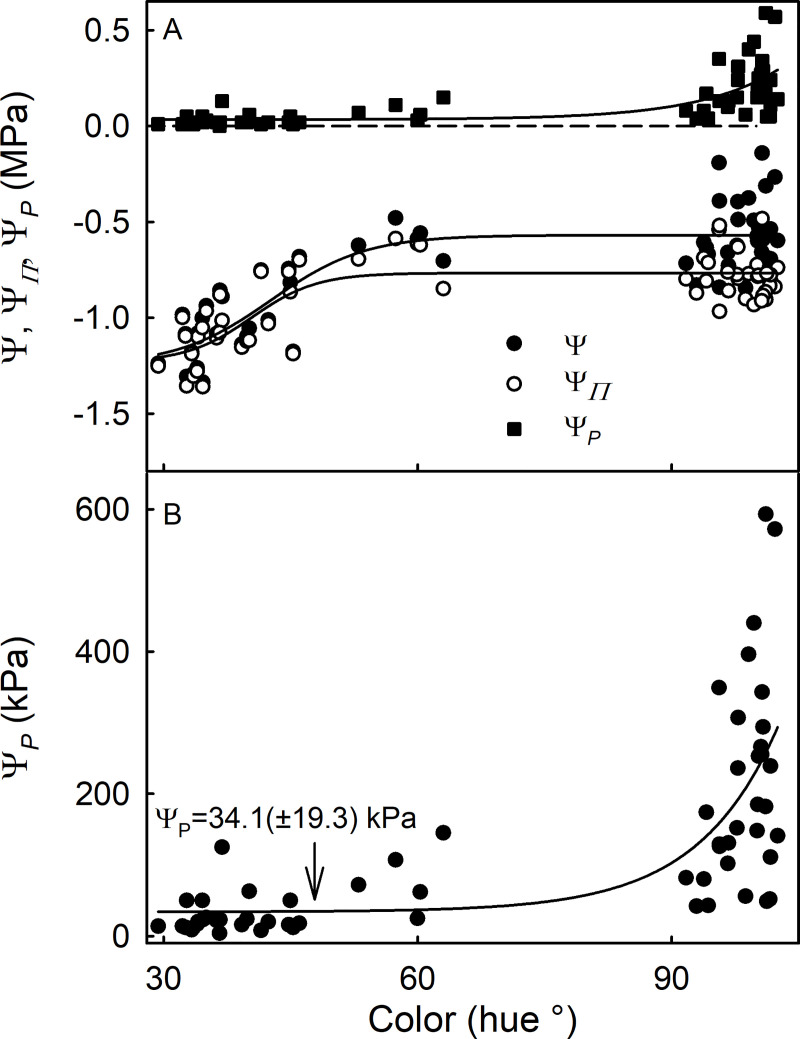
Water potentials in developing strawberries. (A) Calculated fruit water potential (Ψ), osmotic potential (Ψ_Π_) and cell turgor (Ψ_P_) of developing strawberry fruit; (B) Ψ_P_ in A but redrawn on a different scale. The value of Ψ was calculated as Ψ = Ψ_Π_ + Ψ_P_. (Bars represent SE). The arrow indicates the Ψ_P_ at maturity.

The osmotic potential of the expressed fruit juice was nearly constant for unripe fruit ranging from green (hue angle >90°) to white (hue angle ≈ 60°), but decreased and became more negative as the fruit turned red (hue angle <60°) (Fig 3A). Fruit turgor (Ψ_P_) was very low throughout most of the fruit-development period, compared to the negative values of fruit osmotic potential. Only during the early stages of development, when the fruit were still green, was cell turgor pressure significantly higher, when average values were about 200 kPa, with occasional peak values of up to 600 kPa being recorded (Fig 3B).

**Fig 4:**
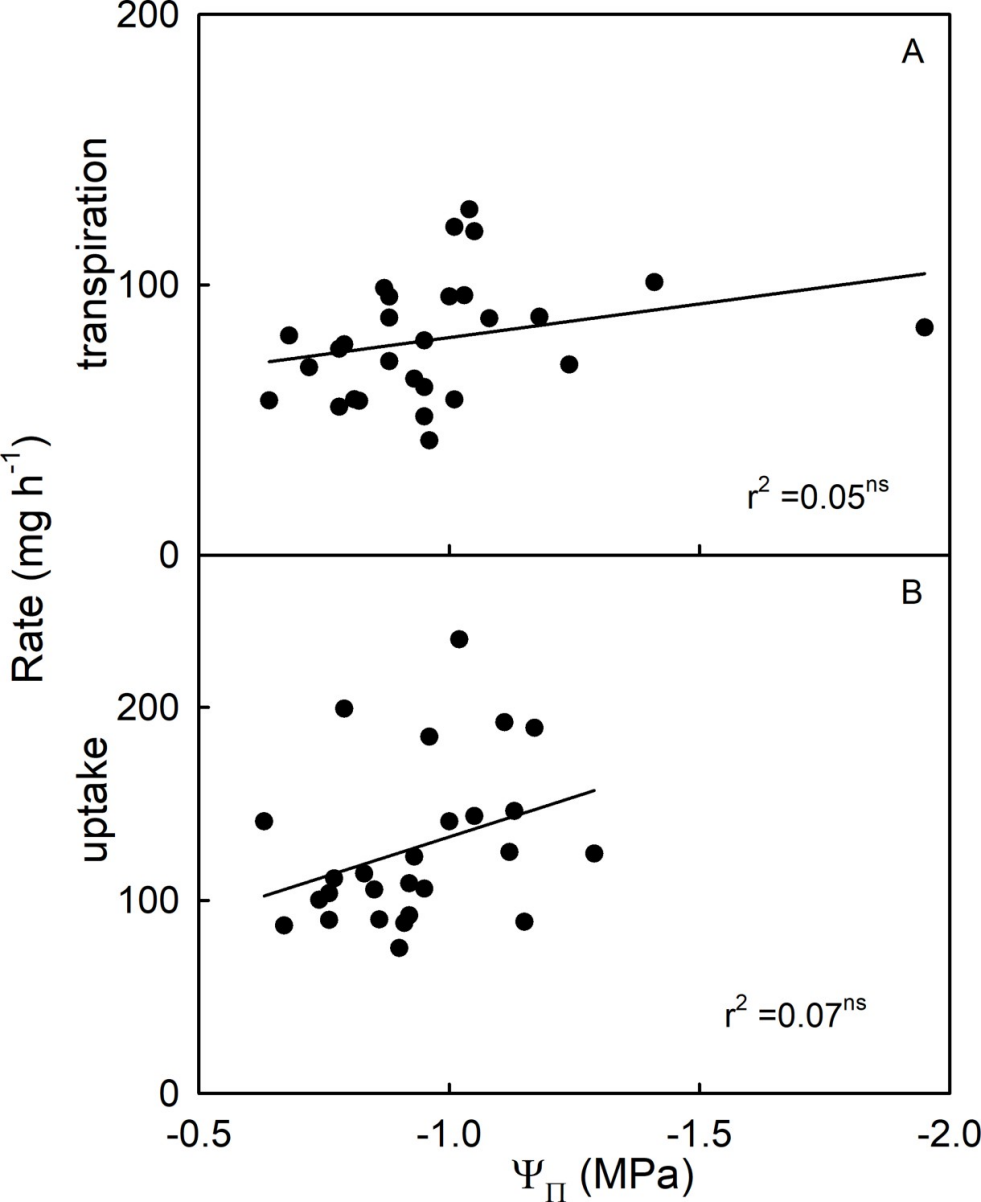
Relationship between osmotic potential (Ψ_Π_) of a strawberry fruit’s expressed juice and water movement. (A) Rates of transpiration and (B) Rate of osmotic water uptake. Coefficients of determination (r^2^) not significant at P < 0.05.

There was no relationship between the rate of transpiration or the rate of uptake and the osmotic potential of expressed juice for fruit on commercial ripeness (Fig 4A and 4B).

**Fig 5:**
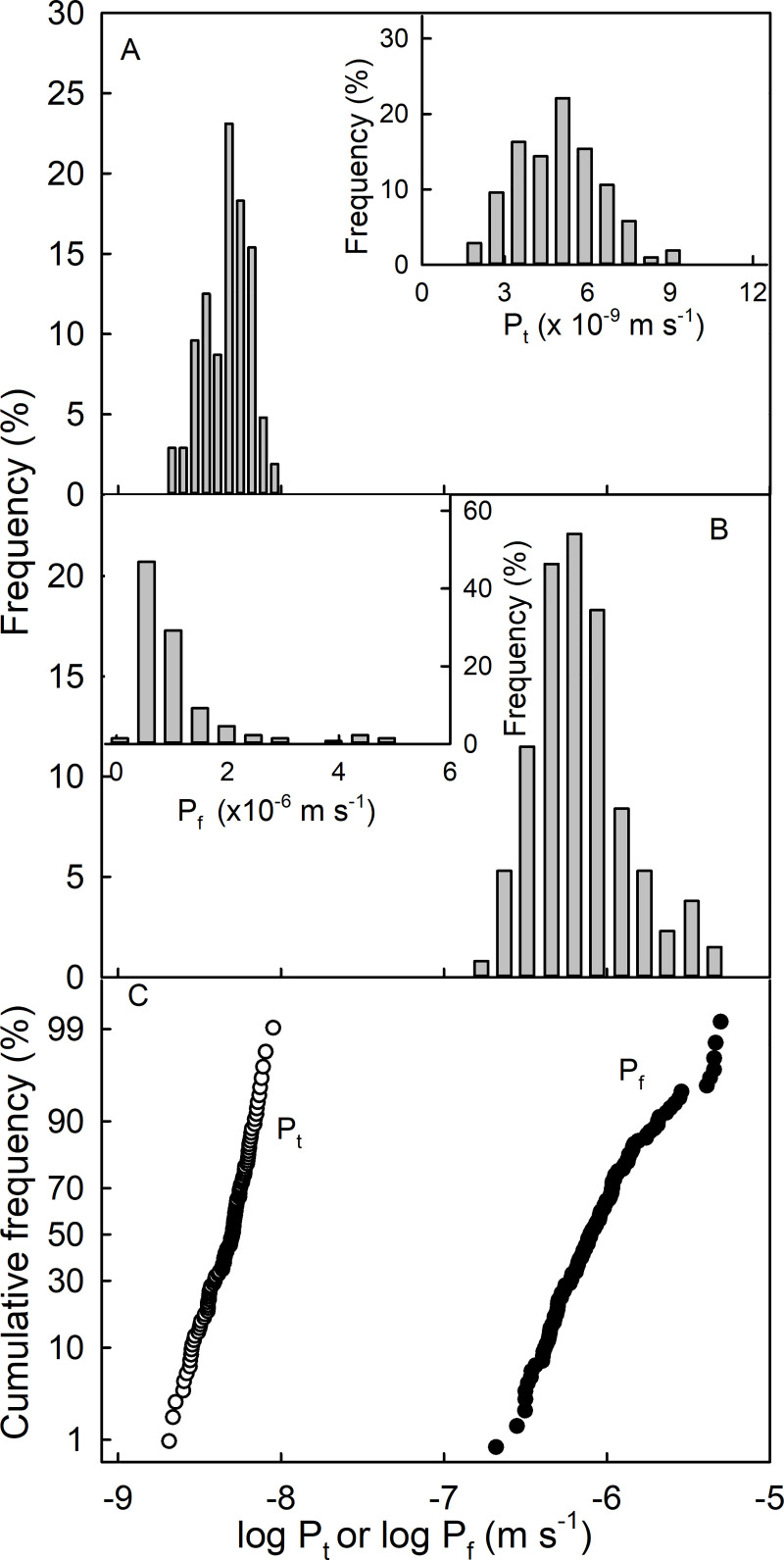
Frequency distributions of skin permeances of strawberries. (A) Log-transformed permeance for transpiration (P_t_) (main graph) and un-transformed P_t_ (inset). (B) Log-transformed permeance for osmotic uptake (P_f_) (main graph) and un-transformed P_f_ (inset). (C) Normal probability plot of the log-transformed permeance of P_t_ and P_f_.

Skin permeances for transpiration (P_t_) and for osmotic uptake (P_f_) follow a log normal distribution as indexed by approximately symmetrical frequency distributions when plotted on a log scale and by linear normal probability plots (Fig 5A–5C). The permeance for osmotic uptake was 226-times larger than that for transpiration (Table 2). This huge dissimilarity is not unique to ‘Clery’ strawberry, but was of similar magnitude in ‘Florentina’ and ‘Laetitia’ (Table 1).

**Table 2:** Permeance for transpiration (P_t_) and permeance for osmotic uptake (P_f_) of the skins of ripe strawberry fruit cv. Clery.

Permeance x 10^−9^ (m s^-1^)	Mean	Median	SE	Range		CV (%)	Number of observations (n)
				Min	Max		
Transpiration (P_t_)	4.9	5.1	0.1	2.1	9.1	30.9	104
Osmotic uptake (P_f_)	1108.0	793.3	86.1	209.1	5104.1	89.0	131

**Fig 6:**
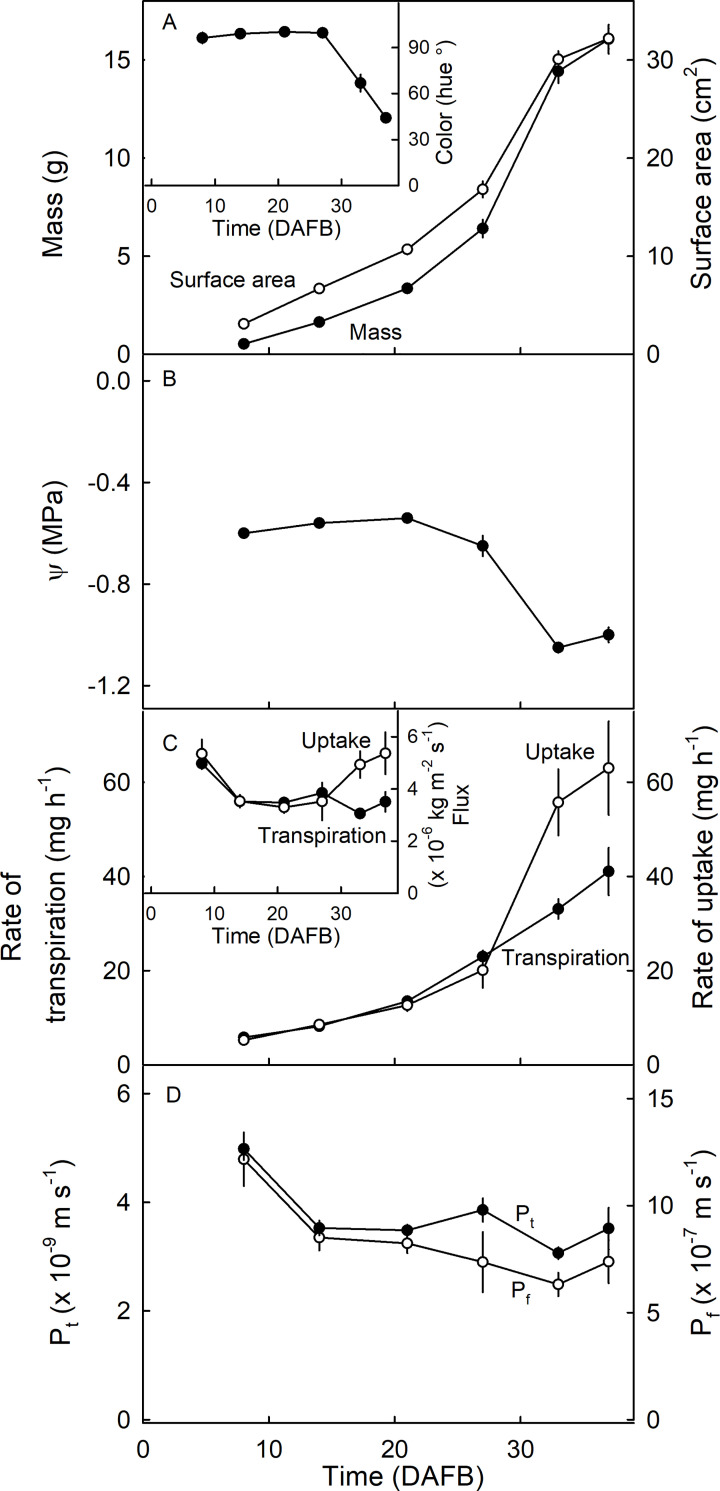
Time course of strawberry development. Change in fruit mass (A), surface area (main graph) and color as indexed by the Hue angle (inset), (B) water potential (Ψ), and (C) flow rates of osmotic uptake and transpiration (main graph) and flux densities of transpiration and osmotic uptake (inset) and permeances of the skin for osmotic uptake (P_f_) and for transpiration (P_t_) (D) (Bars represent SE).

The increase in strawberry fruit mass and, hence, in surface area during development followed a sigmoidal pattern with time (Fig 6A- main graph). Color change as indexed by the decrease in hue angle from green (>90°) to white (≈60°) and finally to fully red (<44°) occurred at about 27 days after full bloom (DAFB) (Fig 6A—Inset). This corresponds to the phase of maximum mass growth rate and of maximum decrease (more negative) in osmotic potential (Fig 6B). Rates of transpiration and of osmotic uptake both increased markedly during development (Fig 6C- main graph). Meanwhile, rates of transpiration (per fruit) decreased during development, while rates of osmotic uptake (per fruit) increased markedly after the fruit turned red (Fig 6C- inset). The permeances (related to flux densities) for transpiration and osmotic uptake decreased as development progressed (Fig 6D). The permeance for osmotic uptake was markedly and consistently higher than that for transpiration—by about two orders of magnitude.

**Fig 7:**
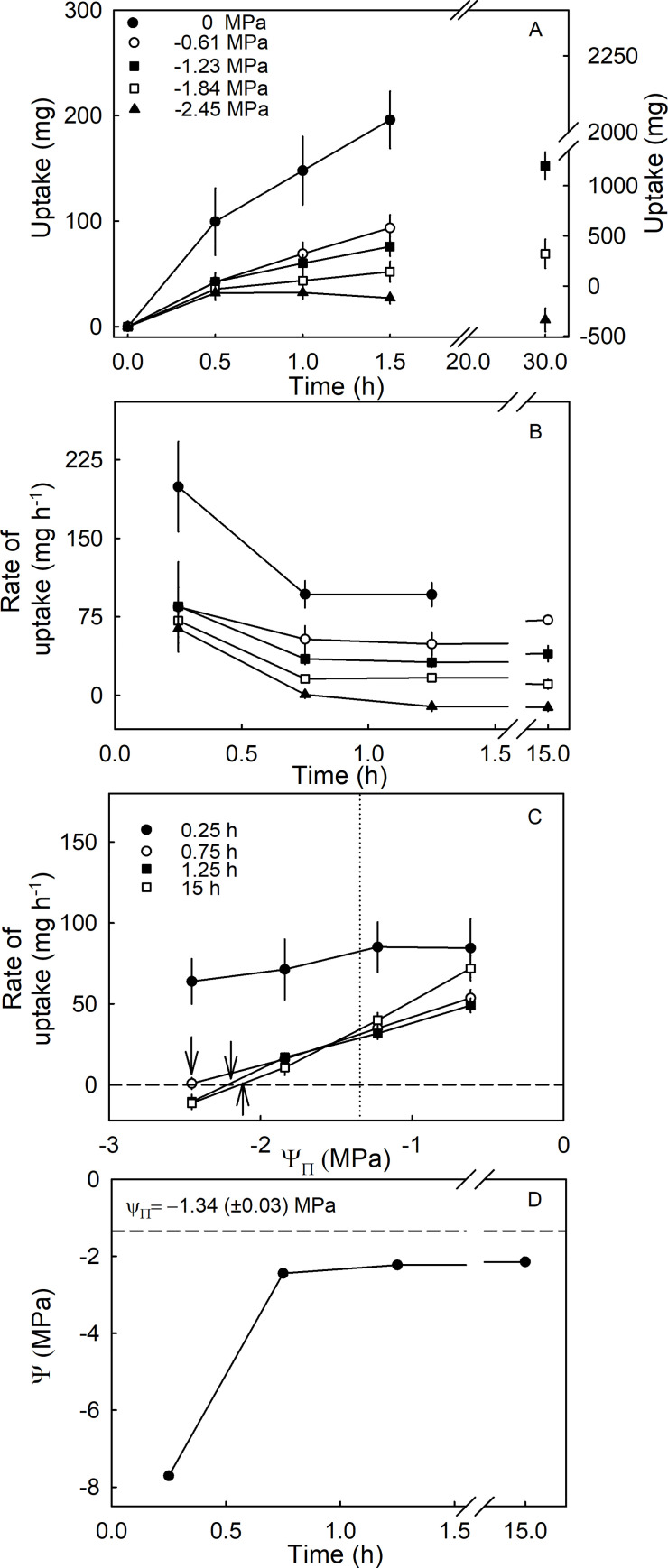
Effect of osmotic potentials (Ψ_Π_) of the incubation solution on osmotic uptake. (A) Time course of cumulative uptake (B) and of change in rate of uptake. (C) Relation between rate of uptake and the osmotic potential of the incubation solution. The arrows indicate the osmotic potential at zero water uptake. At this point the osmotic potential of the incubation solution equals the calculated fruit water potential. The vertical dotted line indicates the osmotic potential of the expressed juice. (D) Time course of change in the calculated fruit water potential. Water potential was estimated from the osmotic potential of the incubation solutions for zero water uptake. (Bars represent SE). Glucose and fructose at a molar ratio of 1:1 were used as osmolytes in the incubation solution because these two carbohydrates represent the most abundant osmolytes in strawberries.

Rates of osmotic uptake depended on the osmotic potential of the incubation solution (Fig 7). Decreasing the incubation osmotic potential decreased the rate of osmotic uptake (Fig 7B). Interestingly, positive rates of osmotic water uptake were recorded from isotonic, and even from hypertonic solutions (Fig 7A). The rates of osmotic uptake were consistently higher during the first experimental interval (0.25 h), before decreasing towards an asymptote and then remaining about constant for up to 30 h (Fig 7B). Fitting a linear regression through a plot of osmotic uptake rate vs. the osmotic potential of the incubation solution, allowed fruit water potential to be estimated from the x-axis intercept. At this point, the driving force for osmotic water uptake is zero because fruit water potential (unknown) equals the osmotic potential of the incubation solution (known). This calculation revealed very negative values for fruit water potential during the first incubation interval. Fruit water potential then gradually increased (became less negative) as it approached the osmotic potential of the expressed fruit juice. The values found for Ψ_fruit_ were consistently more negative than those measured for the osmotic potential of the expressed fruit juice (Ψ_Π_) (Fig 7D). This difference amounted to about -0.6 MPa.

**Fig 8:**
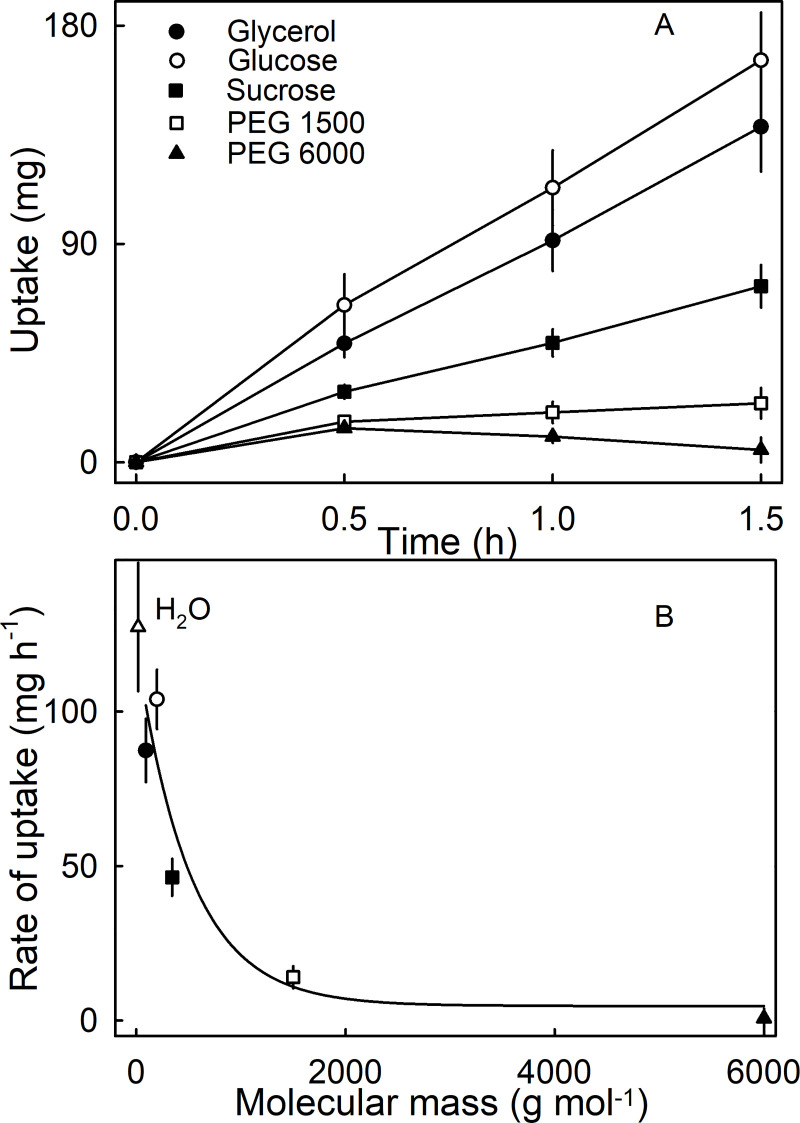
Effect of different molecular mass of isotonic solutions on osmotic uptake. (A) Cumulative uptake. (B) Rates of uptake. (Bars represent SE).

Incubating fruit in isotonic solutions composed of osmolytes of different molar mass, resulted in osmotic water uptake at rates that depended on the molar masses of the osmolytes (Fig 8A). Osmolytes having molar masses of 1500 g mol^-1^ or lower resulted in rates of osmotic uptake of > 0 mg h^-1^. The rate of osmotic uptake decreased asymptotically as molecular size increased (Fig 8B).

**Fig 9:**
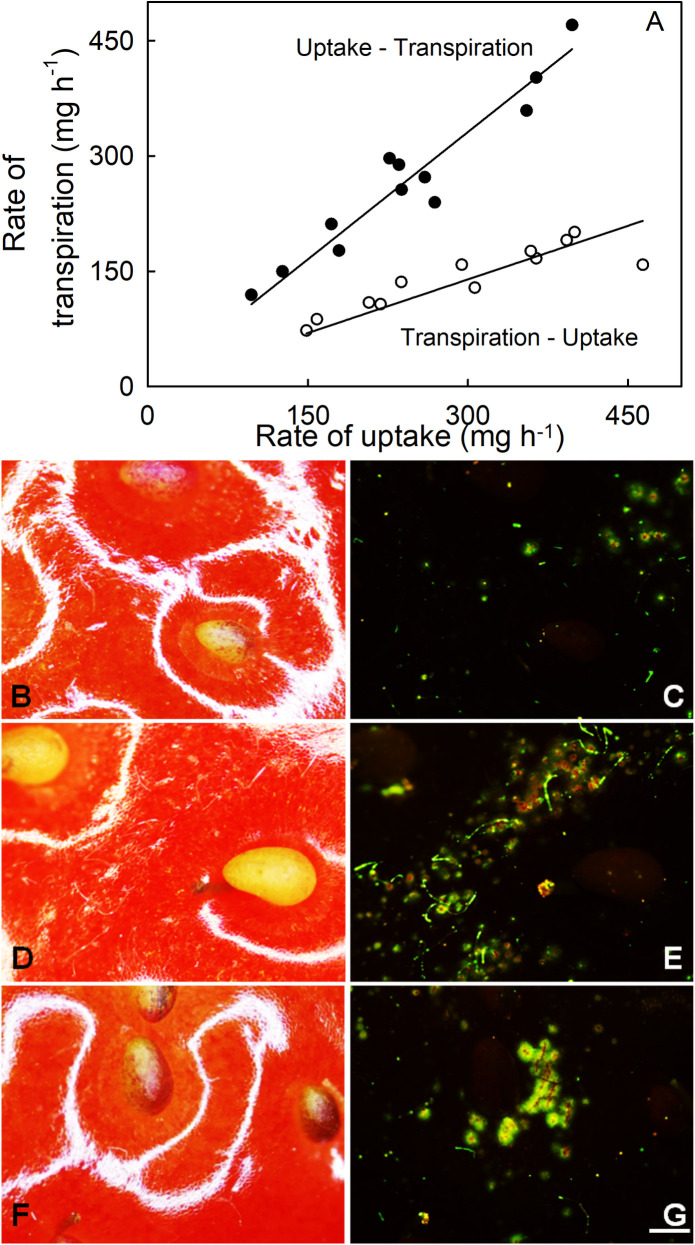
Effect of sequence when determining both water uptake and transpiration on water movement and on formation of microcracks. (A) Relationship between rate of osmotic uptake (F_f_,) and rate of transpiration (F_t_) for fruit subjected to the osmotic uptake determination first, followed by transpiration (‘uptake→transpiration’) and vice-versa (‘transpiration→uptake’). The regression equations were: F_t_ = 1.10 (±0.03) x F_f_, r^2^ = 0.92*** (uptake before transpiration); F_t_ = 0.47 (±0.02) x F_f_, r^2^ = 0.71*** (transpiration before uptake). The intercepts were not significant, so regression lines were forced through the origin. Significance of slope parameter at P < 0.001 indicated by ***. Microscopic view of a strawberry fruit surface under incident bright (B, D, F) and fluorescent light (C, E, G) following incubation in acridine orange solution (0.1%) for 5 min. Fruit was subjected to 1.5 h of transpiration (B and C) or to 1.5 h of osmotic uptake (D to G). Following water uptake, ring microcracking was plainly evident in the epidermis around the achenes (E) and in the achene depressions themselves (G). Scale bar 500 μm.

When conducting transpiration and osmotic-uptake experiments sequentially on the same fruit, the rates of water movement recorded depended on the order in which the experiments were conducted (Fig 9A). That is, the rate of transpiration was consistently higher when recorded after first recording the osmotic uptake rate, and lower if recorded before. Incubating fruit in the fluorescence tracer acridine orange revealed considerable microcracking after fruit were incubated in water (i.e. as per an osmotic-uptake experiment). The microcracks were in the areas of epidermis lying between the achenes and were ring-shaped and centered on the achenes (Fig 9D and 9E) and in the depressions of the achenes (Fig 9F and 9G). In contrast, fruit that had not been incubated in deionized water (i.e. as per a transpiration experiment) had markedly fewer or no microcracks (Fig 9B and 9C).

## Discussion

### Mature strawberries have very low turgor

Low fruit turgor pressure is not unique for mature strawberries but has been reported also in the mature fleshy fruit of many species including of grape berries [26–30], sweet cherry, plum, currents and tomato [18,31]. For strawberries, low turgor pressures are also in agreement with calculations made by [32], who estimated turgor pressures of 0.2 (unripe fruit) to 0.05 MPa (ripening fruit) by subtracting measured fruit osmotic potential from measured fruit water potential.

Low turgor pressures occur in many species of ripe fruit, despite the usually very negative osmotic potential of their expressed juices. As a consequence, in mature fruit, the water potential is essentially equal to the osmotic potential. However, in the unripe fruit of many fruit species, the turgor is usually markedly higher. Our findings for strawberries (pseudocarps) follow this general pattern found for true fruit. Thus, for grape berries, a transient peak in turgor coincides with veraison [33]. In sweet cherry, peak turgors occur at the onset of color change, when rates of fruit growth and rates of accumulation of carbohydrates are at maximum [31].

Accumulation of apoplastic solutes in the cell wall space of grape berries is responsible for the lack of turgor at maturity [33,34]. This is also the case in strawberries. The apoplast of ripe strawberries contains high levels of solutes, but not that of unripe ones [32]. The consequences of this are two-fold: 1) High concentrations of apoplastic solutes generate a negative water potential in the apoplast and the decreased turgor, in turn, facilitates phloem transport and thus phloem water inflow into the fruit. 2) The resulting lack of turgor implies that fruit water potential and osmotic potential are essentially equal and very low (negative) in mature fruit. In sweet cherry, the negative fruit water potential results in water being pulled into the fruit osmotically even in the absence of transpiration [35]. Whether this is also the case in strawberry is currently unknown.

### Strawberry skins are highly permeable to water and their permeance for osmotic uptake very greatly exceeds that for transpiration

When compared with other fruitcrop species, the permeances of strawberry fruit skin recorded here for transpiration and also for osmotic uptake are the highest ever recorded (see compilation in Table 1). Thus, the strawberry skin is not a very effective barrier to the movement of water.

To the best of our knowledge, the surface of a mature strawberry fruit is covered by the thinnest fruit cuticle ever reported (Table 1). There was no significant relationship between cuticle thickness and cuticle permeance. The extremely thin cuticle implies an extreme likelihood that surface defects will result if the fruit skin is subjected to high rates of strain during growth. The intracuticular waxes rather than the epicuticular waxes are considered to form the primary barrier to water movement across plant surfaces [36].

So, an extremely thin cuticle indicates an extreme dearth of intracuticular waxes and thus an extremely water-permeable skin. Moreover, our results reveal numerous cracks in the cuticle, particularly after the strawberry fruit skin has been in contact with surface water. Thus, an extremely thin cuticle along with a predisposition to microcracks indicates the barrier properties of a strawberry fruit’s skin will be very limited in the dry and still further impaired by any exposure to surface wetness.

It is interesting to note that the permeance to osmotic uptake exceeded that to transpiration by more than two orders of magnitude. The average ratio P_f_/P_t_ is about 190 for strawberry. This ratio is much larger than for other fruitcrop species (Table 1) where P_f_ generally exceeds P_t_ but where the ratio is much less extreme (i.e. P_f_/P_t_ is about 4 for skins of grape berries and about 27 for skins of sweet cherries) [19,22]. This observation can be adduced as evidence for the involvement of a viscous flow, syn. mass flow, component in osmotic uptake–i.e. along a pathway with a liquid water continuum [21]. Viscous water flow along a liquid continuum across the lipophilic cuticle is very rapid compared with diffusion of individual water molecules across the cuticle. The latter occurs by sorption to the cuticle, diffusion across the cuticle and desorption of individual water molecules at the innerside of the cuticle. For polar penetrants such as the water dipol, the affinity for the lipophilic cuticle is low [37].

The involvement of viscous flow in osmotic uptake is consistent with the following observations:

1. Microcracks occur on the strawberry surface as indexed by penetration of the fluorescence tracer acridine orange (Fig 9). Simulating microcracking by abrading the cuticle increased the rate of transpiration 2.6-fold and the rate of osmotic uptake 7.9 fold. That microcracks are important in osmotic uptake is also inferred from the ‘sequence effect’ when osmotic uptake and transpiration were determined sequentially—i.e. osmotic uptake before transpiration vs. transpiration before osmotic uptake. Transpiration was markedly increased when osmotic uptake was measured before transpiration. Incubation in water induces cuticular microcracking and so impairs the cuticle’s barrier function and increases transpiration. In grape berries, simulated microcracking increased the rates of water uptake 47-fold compared with non-treated control fruit [38]. Similar data were reported by [39] for sweet cherry where water uptake rates were almost double than those of control fruit.
2. Polar pathways occur in cuticles of some fruit crops [16]. These provide an aqueous continuum across the lipophilic cuticle that allows penetration of polar substances by viscous flow [40,41]. These pathways are not physical holes in the cuticle but polar domains that accommodate polar penetrants [42]. They result from the orientation of polar functional groups in the cuticle [43]. Evidence for the presence of polar pathways in strawberry skin comes from the effect of osmolyte molecular size on the rate of osmotic uptake from isotonic solution. The experiment provides evidence for an increase in osmotic uptake that depends on the molecular size of the bathing osmolyte. Because the osmolytes we chose are polar (and polar osmolytes are excluded from lipophilic pathways) their penetration must have occurred via a polar pathway. And this penetration was size-selective. The smallest non-penetrating osmolyte was PEG 1500 (1500 g mol^-1^) and the largest penetrating osmolyte was sucrose (342 g mol^-1^). The size selectivity of the polar pathways of a strawberry fruit skin is in close agreement with that reported previously [16, 17].

From the above observations we infer that viscous flow through microcracks and polar pathways across the extremely thin cuticle accounts for the high permeability in osmotic uptake as compared to transpiration. This interpretation is consistent with a lower coefficient of determination for the relationship between flow rate and fruit surface area in osmotic uptake as compared to transpiration. Apparently, the occurrence of microcracks and the frequency of polar pathways–both involved in osmotic water uptake–is independent of area. In contrast, transpiration occurs primarily by diffusion across the cuticle and hence, bears a closer relationship with surface area.

## Conclusions

The high permeability of strawberry fruit skins to water is of significant commercial importance. The very high permeability for transpiration (water loss) accounts for the special susceptibility of strawberries to postharvest water loss during handling, transport and shelf life. The initial water loss results in loss of shine which makes the fruit less appealing to the consumer. Further water loss causes visible shriveling. The high permeability for osmotic uptake contributes to the very limited ‘rainfastness’ of strawberries in the field, where unprotected strawberry fruit are highly susceptible to skin cracking and water soaking [1]. It is likely that rapid water uptake and the bursting of cells also contribute to these problems. Further research is needed to identify causal relationships and mechanisms. The findings reported in this paper are an important prerequisite.

## Supporting information

S1 Dataset Excel file containing all data produced in figures and tables throughout the manuscript. (XLSX)
